# Cocaine-Induced Plasma Cell Dermatomucositis (CI-PCDM): Clinical and Histopathological Features of a UK Cohort

**DOI:** 10.1007/s12105-026-01934-y

**Published:** 2026-06-11

**Authors:** Grigorios Thermos, Emily Morrison, Laura Cuddy, Waseem Ghumra, Konstantinos Paraschou, Catherine Brophy, Arun Takhar, Theofano Tikka, Paris Tamiolakis, Nitin Khirwadkar

**Affiliations:** 1 Cellular Pathology, Royal Liverpool University Hospital, University Hospitals of Liverpool Group, Liverpool, UK; 2 Institute of Life Course and Medical Sciences, University of Liverpool, Liverpool, UK; 3 Dermatology, Broadgreen Hospital, University Hospitals of Liverpool Group, Liverpool, UK; 4 ENT/Head and Neck surgery, St George’s Hospital, St George’s University Hospitals NHS Foundation Trust, London, UK; 5 Head and Neck Pathology, Guy’s and St Thomas’ Hospital, Guy’s and St Thomas’ NHS Foundation Trust, London, UK

**Keywords:** Cocaine, Plasma cells, Mucositis, Dermatitis, Levamisole, IgG4

## Abstract

**Background:**

Cocaine-induced plasma cell dermatomucositis (CI-PCDM) is an emerging clinical entity involving ulcerated, nodular lesions of the nasolabial region. While it shares some clinical features with other cocaine-induced midline disorders, CI-PCDM is increasingly recognized as a distinct inflammatory process characterized by specific histological patterns.

**Methods:**

This retrospective, multi-center case series identified six patients from two UK pathology archives. Data were retrieved from electronic records and medical photography, while histopathology and immunohistochemistry reports were reviewed to evaluate diagnostic features and exclude alternative pathologies.

**Results:**

The cohort consisted of five males and one female (mean age 50.3 years). Patients typically presented with exophytic, ulcerated masses in the nasolabial area. Cocaine exposure was confirmed in all cases through clinical history or toxicology. Histology consistently revealed dense, polyclonal plasmacytic infiltrates in perivascular and periadnexal distributions. Serological and immunohistochemical variations were noted, including instances of antibody positivity and altered protein ratios. Management strategies, including medical and surgical interventions, generally resulted in partial clinical improvement.

**Conclusion:**

This first UK-based case series highlights CI-PCDM as an emerging diagnostic challenge. The prominent cellular infiltrate may reflect a chronic, antigen-driven immune response, potentially related to localized cytokine activity triggered by cocaine and/or certain adulterants, such as benzocaine. The clinical presentation often mimics other common mucocutaneous lesions, necessitating thorough clinicopathological correlation. Increased clinical awareness is essential for accurate identification and for establishing optimal management protocols for affected patients.

## Introduction

 Once employed in clinical practice as a topical anesthetic derived from coca plant species, cocaine exerts complex pleiotropic effects, primarily mediated through inhibition of monoamine reuptake and robust activation of the sympathetic nervous system, culminating in both systemic and tissue-specific sequelae [1]. Central to its pathogenicity is intense vasoconstriction, which underlies many of its ischemic and vasculopathic complications [1].

Currently, cocaine is used illicitly, most commonly through intranasal insufflation or inhalation, and has re-emerged as a significant and escalating public health concern in the United Kingdom and across Europe [2, 3]. The latest surveillance data from the Office for National Statistics indicate that approximately 3% of adults aged 16–59 years in the UK report on cocaine use, with a disproportionately high prevalence among young adults aged 16–24 years [3].

As cocaine use continues to rise, and as the drug is increasingly adulterated with various substances that may potentiate or modify its properties, most notably levamisole, an expanding spectrum of atypical clinical presentations are recognized across multiple systems, including the head and neck mucosal and skin surfaces [4]. Classically, most cocaine-related manifestations are attributed to direct cytotoxic effect on local tissues, but it is now understood that others are part of a broader, systemic response to cocaine exposure simulating autoimmune vasculitides with presence of circulating autoantibodies (cocaine and levamisole-induced vasculitis or cocaine-induced pseudo-granulomatosis with polyangiitis-GPA) [4, 5]. Such immunologic activation may coexist with a diverse range of topical mucosal and cutaneous manifestations, spanning necrotic mucosal lesions (cocaine-induced midline destructive lesions-CIMDL), eczema-like eruptions, neutrophilic dermatoses, and prurigo-like lesions.

More recently, a distinct clinicopathological entity has been described in cocaine users who do not commonly show serological or histological evidence of vasculitis or neutrophilic disorder, coined as Cocaine-induced plasma cell dermatomucositis/orificial mucositis (CI-PCDM) [6, 7]. This condition characteristically involves the periorificial region of the upper lip extending to the nasal philtrum and nostrils. Clinically, it presents as a progressively enlarging, exophytic, and partially ulcerated plaque, with features that may closely mimic malignancy [6, 7]. As its name suggests, it is characterized microscopically by a dense, polyclonal plasma-cell–rich infiltrate, often accompanied by variable eosinophilia [6, 7].

In this case series, we report six new cases of cocaine-induced plasma cell dermatomucositis, highlighting their clinical presentation and histopathological features aiming to increase awareness of this rather underreported entity.

## Materials and Methods

This retrospective, multi-center case series identified patients diagnosed with CI-PCDM through a search of the authors’ (N.K. and P.T.) pathology archives and clinical databases.

Clinical data, demographics, further investigations, treatment and follow-up where available were retrieved from electronic records and medical photography. Histopathology reports were reviewed to confirm the diagnostic features and the exclusion of other etiologies.

The study adhered to the 1964 Declaration of Helsinki and complied with the UK Data Protection Act and GDPR and NHS Research Ethics Committee (REC) guidelines. Written informed consent was obtained from three patients for the use of clinical photography; all other data were anonymized to protect patient confidentiality.

## Results

**Fig. 1:**
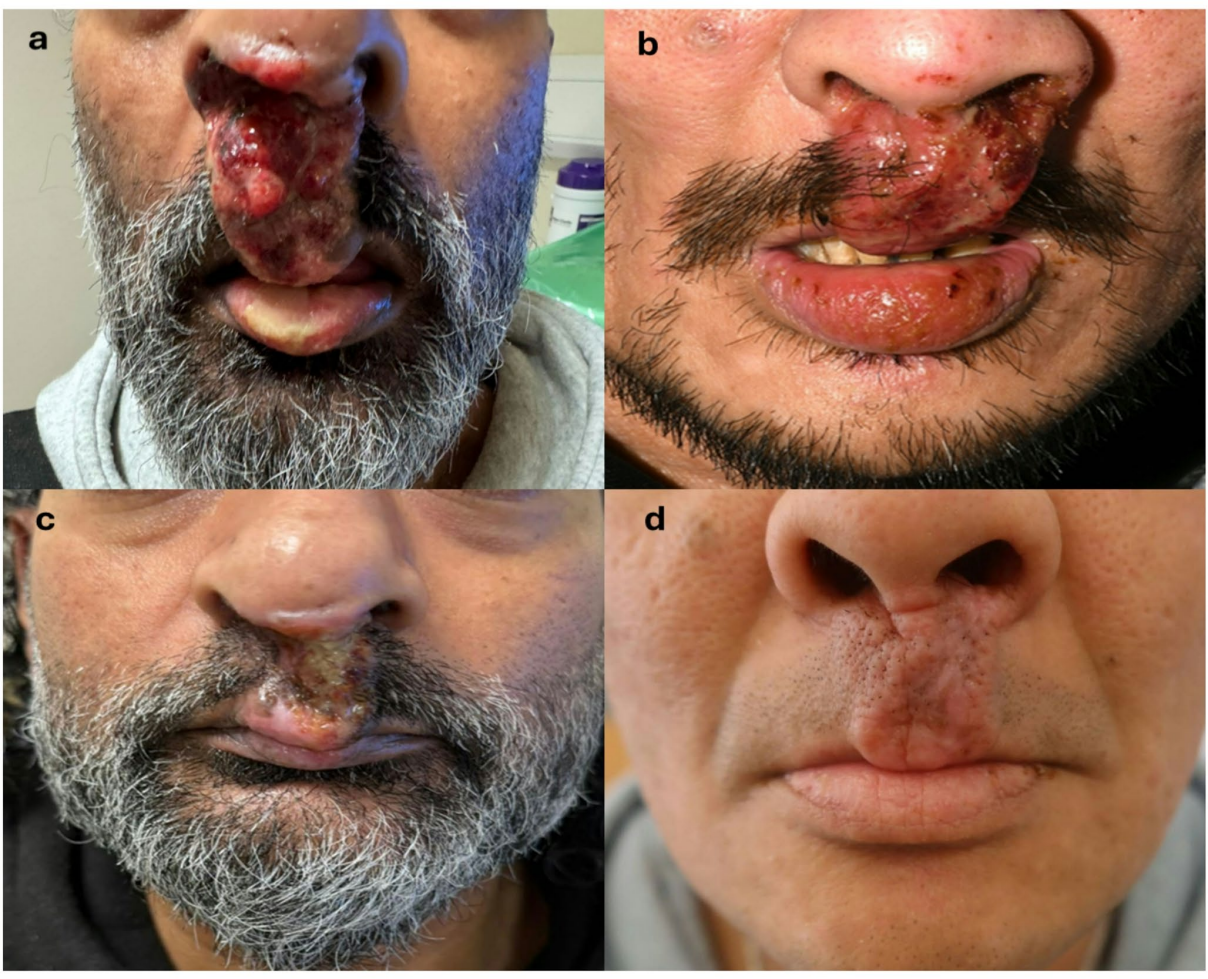
**a–d**: Representative clinical images of CI-PCDM: Variously ulcerated, exophytic plaques affecting the nasolabial region (**a**-Case 1, **b**-Case 4). Follow-up images of Case 1 after surgical debulking (**c)** and Case 4 following management with systemic steroids and cocaine cessation (**d)** indicate partial improvement

A total of six cases were retrieved (Table 1). There was a male predominance (5 males, 1 female) with a mean age of 50.3 years. The most frequent clinical presentation was a variously nodular, exophytic mass associated with ulceration and crusting (Fig. 1a, b) with a mean duration of 7 months. In five of the six cases, the upper lip and nostrils were simultaneously affected; two of these patients also presented with septal perforation. Imaging performed did not demonstrate sinus involvement or other destructive lesions.

**Table 1:** Relevant clinicopathological details of the CI-PCDM cohort

Age/Sex	Clinical presentation	Affected sites	No of biopsies prior to diagnosis	Duration	Histopathological examination	Further investigations	Treatment	Outcome	Medical history
49/M	Large, ulcerative exophytic mass with bleeding	upper lip, tip of nose	4	12 months	Polyclonal dense plasma-cell infiltrates admixed with eosinophils Negative fungal, viral and bacterial stains IgG4/IgG < 40%	IgG4 +, Anti-PR3+ (c-ANCA) Urine analysis (+) for cocaine and cannabis	Surgical debulking	Partial resolution	psoriasis cocaine use (stopped 1 year ago)
43/M	Laceration and septal perforation	upper lip, nostrils, septum	N/A	N/A	Polyclonal dense plasma-cell aggregates admixed with eosinophils, and histiocytes Negative fungal, viral and bacterial stains IgG4/IgG > 40%	Negative serological tests Staphylococcus aureus (+)	N/A	Lost to follow-up	cannabis use suspected cocaine use
47/M	Ulcerated, nodular mass Lymphadenopathy	upper lip, nostrils	1	3 months	Perivascular and perifollicular dense polyclonal plasma-cell infiltrates with numerous eosinophils Negative fungal, viral and bacterial stains IgG4/IgG < 40%	Negative serological tests	Prednisone Cocaine cessation	Partial resolution	JAK2 (-) erythrocytosis Daily cocaine use
49/M	Erythematous mass	upper lip, left nostril	2	6 months	Perivascular and perifollicular dense, polyclonal plasma-cell infiltrates, Negative fungal, viral and bacterial stains IgG4/IgG < 40%	Anti-PR3+ (c-ANCA) Decreased renal function	Prednisolone Cocaine cessation	Partial resolution (residual scar)	Daily cocaine use
51/F	Ulcerated nodular mass with necrotic surface Septal perforation	Right upper lip, right nostril, septum	N/A	5 months	Psoriasiform acanthosis and spongiosis Perivascular plasma-cell and lymphocytic infiltrations Negative fungal, viral and bacterial stains IgG4/IgG < 40%	Negative serological tests Increased kappa/lambda on blood Urine analysis (+) for cocaine	N/A	N/A	Crohn’s disease under adalimumab Autoimmune gastritis Chronic opiate use
63/M	Ulcerated plaque	Nasal philtrum, columella, nasal vestibule (including alar cartilages) septum	1	9 months	Polyclonal dense plasma-cell infiltrates admixed with eosinophils Negative fungal, viral and bacterial stains IgG4/IgG < 40%	Negative serological tests	Cocaine cessation Topical nasal emollients	Under surveillance	Cocaine use

M, male, F, female, N/A, not available

**Table 2:** Review of all CI-PCDM cases published in the literature

References	Age/sex	Clinical presentation	Affected sites	Duration	Histopathology	Further investigations	Treatment	Outcome	Comment
Udondo González del Tánago et al. [8]	60/M	Exudative ulcerated lesion	Upper lip, Left nasal vestibule	1 month	Numerous eosinophils with few eosinophils Negative fungal, viral, bacterial stains	N/A	30 mg of prednisone	Resolution	Originally termed “cocaine-induced plasma cell orificial mucositis”
Viedma-Martinez et al. [6]	45.5 (mean), 6 males, 4 females	Pseudo-tumoral ulcerated plaque	Upper lip, Nostrils	9 months (mean)	Dense plasmacell infiltrate with eosinophils Negative fungal, viral, bacterial stains	c-ANCA (+), (2 cases) ↑ eosinophils (5 cases)	Topical and systemic steroids	Resolution (6 cases) Relapse (1 case)	Originally termed “cocaine-induced plasma cell orificial mucositis”
Fernandez-Flores et al. [7]	56/M	Progressive thickened plaque	Upper lip Nostril	12 months	Neutrophilic infiltrate in upper dermis Deeper plasma-cell infiltrates with eosinophils Negative fungal, viral, bacterial stains	Negative serological tests	N/A	N/A	Proposed the term “cocaine-induced plasma cell dermatomucositis”
Reyes Albaladejo et al. [9]	40/F	Exudative plaque	Supralabial region, nasal vestibule and distal nasal tip	3 months	Dense lymphoplasmacytic infiltrate IG4:IGG > 40% Negative fungal, viral, bacterial stains	↑ neutrophils, ↑ eosinophils, c-ANCA (+)	45 mg of prednisone	Resolution	
Tordjman et al. [10]	66/M	Ulcerated plaque	Right nasolabial region	7 months	Dense plasma-cell infiltrate with eosinophils S.Aureus and P.Aeruginosa (+)	Negative serological tests	Prednisone	Recurrence upon tapering	
Patel Housley et al. [11]	53.6(mean) 2 males, 1 female	Vegetative plaques	Upper lip,nasolabial folds	2 months (mean)	Dense plasma-cell infiltrate with eosinophils	c-ANCA (+) all cases	Rituximab (1 case) Systemic steroids (1 case)	Resolution (2 cases)	Uses the term “cocaine-induced plasma cell orificial mucositis
Our case series	50.3 (mean) 5 males, 1 female	Uclerated plaque Septal perforation (2 cases)	Upper lip, nostrils (all cases) Septum (2 cases)	4.8 months (mean)	Dense plasma-cell infiltrate with eosinophils Negative fungal, viral, bacterial stains S.Aureus (+) (one case) IgG4:IgG ~ 60% (one case)	c-ANCA (+) (2 cases), IgG4 ↑ (one case) kappa, lambda ↑ (one case)	Surgical debulking (1 case) Predinisolone (2 cases) Topical nasal emollients (1 case)	Partial resolution (3 cases)	
Summary:	45.5 (Viedma-Martinez et al.2024)* 52.7 (rest of cases)	Variably ulcerated plaque/lesion (23 cases) Septal perforation (3 cases)	Upper lip and nasal cavity (23 cases) Septum (3 cases)	5.6 months (mean; all cases)	Deeper plasma-cell infiltrates (23/23) IG4:IGG > 40% (2/20) S.Aureus and P.Aeruginosa (+) (2 cases)	c-ANCA (+) (8/23) ↑ eosinophils (3 cases) Kappa, lambda ↑ (one case) IgG4 ↑ (one case)	Systemic and topical steroid treatment Surgical debulking Topical nasal emollients	Variable results	

*Does not state exact age of patients

M, male, F, female, N/A, not available

Illicit drug use was reported in all cases. While four patients admitted to cocaine use, two initially denied it; however, one of the latter subsequently tested positive for cocaine via urinalysis. 

**Fig. 2:**
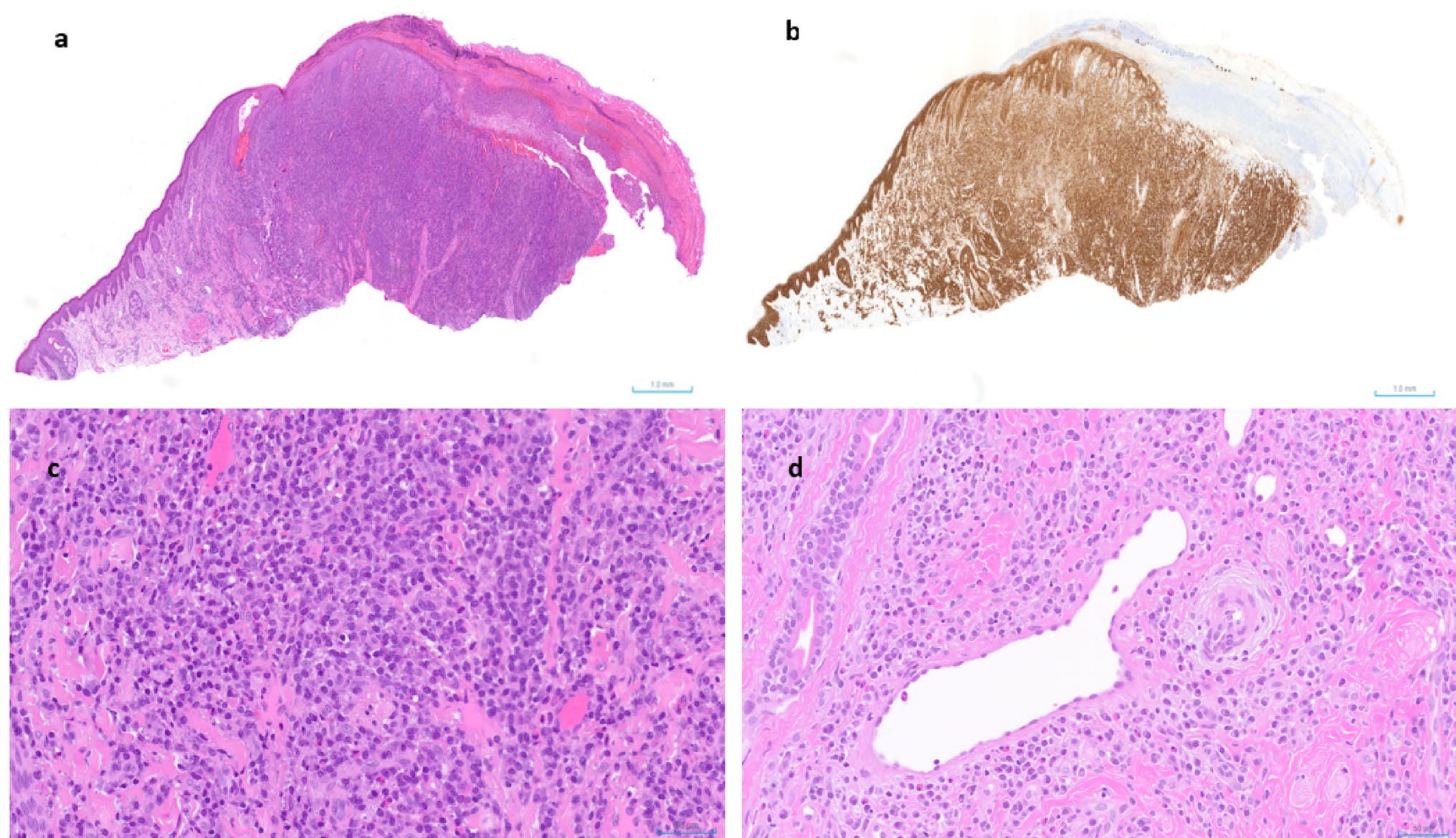
Representative histopathological images of CI-PCDM: **(a)** exophytic, partly ulcerated plaque showing dense, deep seated plasma-cell infiltrates. **(b)** Diffuse positivity for plasma cell marker CD138 (**c**) There are numerous plasma cells admixed with eosinophils arranged in perivascular and periadnexal distribution. (**d**) No evidence of necrosis or vasculitis is seen

**Fig. 3:**
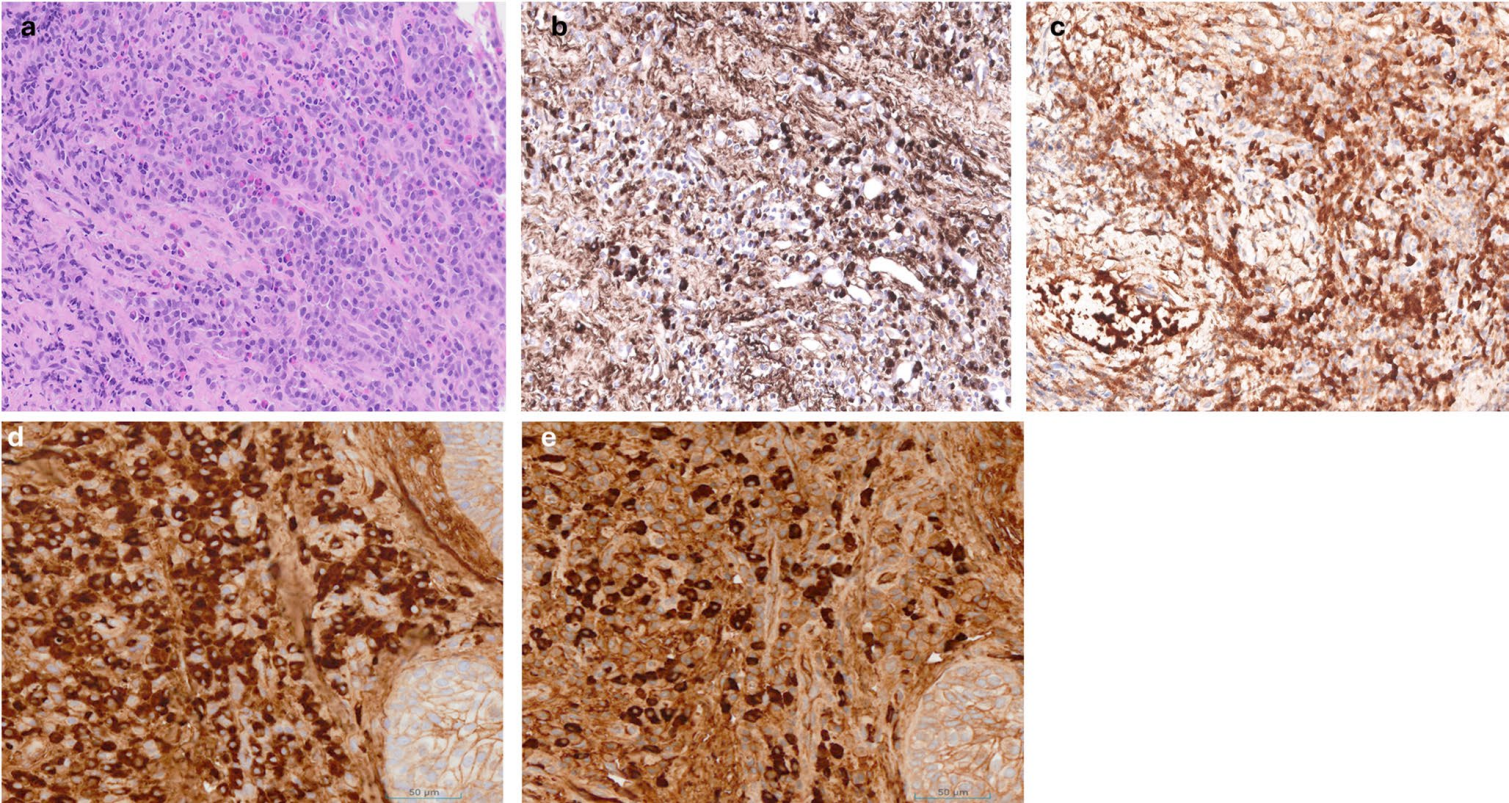
Representative immunohistochemistry images of CI-PCDM: One case showed increased IgG4:IgG ratio; hematoxylin and eosin (**a**), IgG4 (**b**) and IgG (**c**) (original magnification: 10x), Staining for kappa (**d**) and lambda (**e**) light chains revealed a polyclonal plasma-cell population

In two cases, multiple biopsies were performed before reaching a final diagnosis. On histopathology, all cases demonstrated a dense plasma cell infiltrate within the dermis, typically in perivascular and periadnexal distributions (Fig. 2a–d). No evidence of vasculitis or granuloma formation was observed. In 50% of the cases, a significant number of eosinophils were noted within the plasmacytic infiltrates. Viral, fungal, and bacterial stains were negative across all cases. Immunohistochemical analysis for kappa and lambda light chains confirmed the polyclonality of the plasma cells; in one case, an increased IgG4:IgG ratio was identified (Fig. 3a–e).

All patients underwent serological screening for autoimmune vasculitides. Two patients tested positive for c-ANCA (anti-Proteinase 3-PR3), while no other autoantibodies were detected. Interestingly, one of the seropositive patients subsequently developed a decline in renal function and is currently undergoing investigation for systemic involvement. Regarding IgG4 expression, one patient exhibited an increased serum IgG4:IgG ratio without a corresponding increase in the examined tissue. Another patient demonstrated elevated serum levels of both kappa and lambda light chains, though the ratio remained normal.

In cases where treatment and follow-up data were available, two patients were managed with systemic corticosteroids and one underwent surgical debulking. All patients showed partial clinical improvement, though complete resolution of the lesions was not achieved (Fig. 1c, d).

## Discussion

This is the first case series of CI-PCDM in the UK. To date, only 17 cases (excluding ours) have been reported in the literature; 13 cases have originated from Spanish institutions and 4 cases from the U.S (Table 2) [6–11]. The mean age and gender profile of our cohort are consistent with the existing literature, as are the clinical presentations, affected sites, and histopathological findings.

While it is tempting to call CI-PCDM rare, given the scarcity of published cases, we believe that it probably represents an underreported and underdiagnosed rather than rare entity. This may be because similar nasolabial lesions were not initially biopsied and were “lumped” under the general descriptive term “cocaine-induced midline necrotic lesions-CIMDL”, especially when there was concurrent septal collapse, palatal perforation or other necrotizing mucosal or cutaneous lesions [4]. Additionally, even when biopsy is performed, histopathological features can be non-specific if the deeper dermis is not included in the specimen or may be overlooked by pathologists unfamiliar with CI-PCDM. Moreover, an initial biopsy showing non-specific findings is not an uncommon phenomenon in cocaine-induced disorders such as CIMDL, necessitating multiple biopsies, and careful clinicopathological correlation [12]. Notably, in two of our cases multiple biopsies of the lesions were performed previously before a final diagnosis of CI-PCDM was made, most of them reported as “non-specific inflammation”.

Most patients in our cohort reported frequent cocaine use. One exception was a patient who was a habitual cannabis user but denied using cocaine; another patient, who had a history of using various opiates, tested positive for cocaine on urine analysis disproving their initial statement of abstaining from cocaine use. These instances underscore that confirmatory test for cocaine use, including urinalysis, should be performed in patients presenting with ulcerative nodular lesions in the nasolabial area, particularly when other illicit drug use is reported. A similar approach has been suggested for patients with midline destructive lesions and serological positivity for ANCA to confirm cocaine-induced pseudovasculitis and exclude other autoimmune vasculitides [13].

Interestingly, positivity for cANCA was seen in 6 out of the 17 published cases while 2 cANCA positive cases were found in our cohort. Anti-neutrophil cytoplasmic antibody (ANCA) positivity is reported in up to 70% of CIMDL cases [14]. However, no difference in clinical, serological or histopathological findings between ANCA-positive and ANCA-negative cocaine users that presented with CIMDL was found [14].

The presence of c-ANCA or p-ANCA positivity, paired with septal perforation or necrosis, is a feature also seen in two of our cases, raising the question of whether CI-PCDM belongs to the clinical spectrum of CIMDL and/or cocaine-induced pseudo-GPA [5, 12]. We argue that CI-PCDM is a distinct entity due to its clinical presentation as an isolated, ulcerated tumefactive plaque , likely at the site of cocaine contact, as opposed to the generalized necrotic lesions distant from the nasolabial area seen in CIMDL [5, 12]. Histologically, CI-PCDM is characterized by a predominance of plasma cells and eosinophils without evidence of vasculitis or tissue necrosis. While plasma cells can be found in CIMDL, they are rarely a prominent feature [5, 13]. Nevertheless, the possibility that CI-PCDM is a precursor to, or a subset of, the CIMDL spectrum cannot be entirely excluded given the limited number of reported cases and follow-up data.

The most common cocaine adulterant worldwide, levamisole, exacerbates cocaine-induced tissue destruction by promoting neutrophilic elastase secretion thus inducing neutrophilic infiltration of tissues leading to vasculitis/necrosis and further influencing the ANCA positivity seen in these patients [15]. It remains unclear whether the seropositive patients in our cohort or the literature used cocaine adulterated with levamisole. Notably, in most of our cases, neutrophilic infiltration was not a prominent feature, unlike other levamisole-induced skin lesions [4].

Based on affected site and the histological presence of plasma-cell infiltrates, it is not surprising that CI-PCDM was initially linked to plasma cell mucositis (PCM) or idiopathic lymphoplasmacellular mucositis-dermatitis, the mucosal-cutaneous analogue of Zoon’s balanitis [16, 17]. However, this entity shows primarily mucosal involvement appearing as erythema or cobblestone-like lesions of the gingiva and inner labial mucosa and rarely extends “upwards” to the skin of nostrils [16]. Its exact causes are unknown but is thought to represent a hypersensitivity reaction to an intraoral irritant, such as chewing gum or toothpaste [16]. The histology is strikingly similar to CI-PCDM while some PCM cases have also shown positivity for circulating autoantibodies. Since many of the clinicopathological findings of PCM are identical to CI-PCDM, one might wonder if CI-PCDM is the cutaneous counterpart of PCM, potentially triggered by a cutting agent other than levamisole acting as a local irritant as other authors have previously suggested [6].

Another commonly reported adulterant of cocaine, especially in the UK, is benzocaine, a topical ester anesthetic used to enhance the numbing sensation associated with cocaine [18]. Benzocaine-associated allergic contact dermatitis, contact stomatitis, and other mucosal hypersensitivity reactions are well described [19–21]. In addition, plasma-cell-rich inflammatory infiltrates have been reported in chronic contact hypersensitivity reactions and mucosal inflammatory disorders [22]. In this context, the marked plasmacytosis observed in CI-PCDM may suggest a chronic, antigen-driven immune response distinct from the acute neutrophilic-mediated tissue destruction typical of classic cocaine-induced necrosis. This process may reflect a localized cytokine milieu enriched in Interleukin-6 (IL-6), a cytokine known to promote B-cell differentiation into mature plasma cells [23]. Experimental and clinical studies of contact hypersensitivity reactions have further demonstrated the involvement of IL-6 and related pro-inflammatory cytokines in allergen-driven mucocutaneous inflammation [24]. Repeated intranasal exposure to cocaine adulterated with benzocaine could therefore plausibly act as a persistent local haptenic stimulus, triggering chronic mucosal hypersensitivity and contributing to the plasma-cell-rich inflammatory pattern observed in CI-PCDM [6, 16, 19–25]. Nevertheless, the precise adulterants involved remain uncertain, as specific adulterant analyses were not available in our cohort and have not been systematically reported in previously published cases.

An increased IgG4:IgG ratio in tissue or serum was observed in two of our cases, consistent with other published reports. Reyes Albaladejo et al. [9] postulated that an increased IgG4 ratio can be part of the CI-PCDM spectrum, particularly in the absence of other features of IgG4-related disease (IgG4-RD). The 2020 revised criteria for IgG4-RD combine clinical findings, most commonly internal organ involvement, with serological evidence of IgG4 overexpression (> 135 mg/dl) and specific histological features i.e. storiform fibrosis, obliterative phlebitis and IgG4: IgG ratio of over 40% [26]. While some of the criteria were met in the two previously mentioned cases, they did not qualify for a definitive IgG4-RD diagnosis. Furthermore, increased IgG4 + plasma cells are seen in many other conditions, including autoimmune vasculitides, especially when affecting the head and neck [27]. Interestingly, one patient had an increased tissue IgG4:IgG ratio despite normal serum levels, while another with elevated serum IgG4 did not show an abnormal ratio in the excised tissue, suggesting a discrepancy between systemic and localized IgG4 expression.

Given that increased IgG4-positive plasma cells may occur in a variety of chronic inflammatory and autoimmune conditions and are not specific for IgG4-related disease, it is possible that the IgG4 overexpression observed in a subset of CI-PCDM cases reflects a secondary immune response to chronic antigenic stimulation/chronic inflammation rather than true IgG4-related disease, as various authors have suggested [27, 28].

The differential diagnoses of plasma-cell rich tumefactive plaques in the upper lip and nasolabial skin include a wide range of entities. The polyclonal character and non-atypical appearance of the dense plasma-cell infiltrates rule out extramedullary involvement by plasma cell lymphoproliferative disorders [29]. Exclusion of primary or secondary syphilis is the first step of the diagnostic process and can be easily done by performing relevant histochemical and immunohistochemical stains [30]. Other infections, such as tuberculosis, rhinoscleroma, blastomycosis, or mucocutaneous leishmaniasis, that might affect the nasolabial region in the form of exudative, variously ulcerated plaques, can be excluded histologically by the absence of granuloma formation or bacteria-filled histiocytes, and negative PAS or Grocott-Gomorri stains [31]. One of our cases tested positive for Staphylococcus aureus, likely representing secondary colonization rather than primary infection, as typical signs like suppuration were absent.

From a clinical perspective, CI-PCDM can mimic basal cell carcinoma and squamous cell carcinoma, necessitating urgent biopsy. Indeed, most basal cell carcinomas of the head and neck skin develop in the nose and in more advanced stages they can appear as ulcerative nodular plaques with indurated borders and local tissue destruction (“rodent’s ulcer”), identical to the lesions seen in our patients [32]. Thus, it is prudent to exclude malignancy by histopathological examination even in cases where CI-PCDM is strongly suspected clinically.

Treatment in the literature often involves corticosteroids or immunomodulatory agents, with conflicting results and high recurrence rates [6]. In our cohort, two patients received systemic prednisolone, one was treated symptomatically with nasal emollients, and one underwent surgical debulking. Steroids and surgery provided a partial response, mostly aiding in decrease of the lesions’ size with or without residual scar, but complete resolution of the lesions was not seen. Response to treatment can also be hindered by maintenance of cocaine use during this time, highlighting the necessity of a multidisciplinary approach, including addiction specialists, to achieve long-term remission.

Surgical debulking of CI-PCDM lesions has not been previously reported in the literature. The rationale behind such management in one of our cases was guided by both diagnostic uncertainty and disease burden. Given the extensive lesion size and inconclusive initial biopsy findings, a more comprehensive tissue sample was required to exclude malignancy before initiating any form of immunomodulatory therapy. This was also influenced by the need for anatomical structure preservation in a cosmetically sensitive area such as the nasolabial fold as well as for alleviation of symptoms with regression of the residual inflammatory process.

This study is limited by its retrospective nature and the relatively small number of cases, reflecting the rarity and likely underrecognition of CI-PCDM. In addition, clinical follow-up and ancillary investigations were not uniformly available across all patients due to differences in institutional work-up pathways and tissue availability.

In summary, this two-center case series aims to increase awareness among clinicians and pathologists of this emerging entity when evaluating ulcerated nodular lesions affecting the nasolabial region in patients with a history of cocaine use. Greater recognition and reporting of such cases will facilitate a deeper understanding of its pathogenesis and help define optimal management strategies.

## Data Availability

No datasets were generated or analysed during the current study.

## References

[1] Roque, Bravo, On Behalf Of The Oemonom Researchers et al (2022) Cocaine: An Updated Overview on Chemistry, Detection, Biokinetics, and Pharmacotoxicological Aspects including Abuse Pattern. Toxins (Basel) 14(4):278. PMID: 35448887; PMCID: PMC9032145 35448887 10.3390/toxins14040278 PMC9032145

[2] Lhosmot-Marquet M et al (2026) Cocaine use in Europe: the need for cross-sectoral collaboration between security, justice, health, and social systems. *Lancet Public Health*. ;11(1):e61-e68. 10.1016/S2468-2667(25)00283-X. PMID: 41500743 10.1016/S2468-2667(25)00283-X 41500743

[3] Office for National Statistics. Drug misuse in England and Wales: year ending March 2025 [Internet]. London: Office for National Statistics (2025) Dec 11 [cited 2026 Mar 5]. Available from: https://www.ons.gov.uk/peoplepopulationandcommunity/crimeandjustice/articles/drugmisuseinenglandandwales/yearendingmarch2025

[4] Moreno-Artero E et al (2018) Mucocutaneous manifestations of cocaine abuse: a review. J Eur Acad Dermatol Venereol 32(9):1420–1426. 10.1111/jdv.14912 29512202 10.1111/jdv.14912

[5] Subesinghe S et al (2018) Cocaine and ANCA-associated vasculitis-like syndromes. Autoimmun Rev 17(1):73–79. 10.1016/j.autrev.2017.10.005 29108823 10.1016/j.autrev.2017.11.011

[6] Viedma-Martinez M et al (2024) Retrospective case series of cocaine-associated plasma cell orificial mucositis. JAMA Dermatol 160(3):320–327. 10.1001/jamadermatol.2023.5692 38265770 10.1001/jamadermatol.2023.5692 PMC10809139

[7] Fernández-Flores A, González Montero JM (2024) Cocaine-induced plasma cell orificial dermatomucositis: a more accurate descriptive term for a clearly dermatological entity. Am J Dermatopathol 46(5):305–308. 10.1097/DAD.0000000000002684 38513123 10.1097/DAD.0000000000002684

[8] Del Udondo González B, Jiménez-Gallo D, Navarro-Navarro I, Catalina-Fernández I, Ríos-Martín JJ, Linares-Barrios M (2023) Cocaine-induced plasma cell orificial mucositis. J Eur Acad Dermatol Venereol 37(2):e244–e245. Epub 2022 Oct 14. PMID: 36177500 36177500 10.1111/jdv.18619

[9] Reyes Albaladejo F, Mansilla-Polo M, Botella-Estrada R (2025) Cocaine-Induced Dermatomucositis: Clarifying Diagnostic Controversies and Introducing IgG4 Positivity as a New Histopathological Feature. Dermatol Ther (Heidelb) 15(11):3125–3142. Epub 2025 Aug 19. PMID: 40830329; PMCID: PMC12549497 40830329 10.1007/s13555-025-01512-0 PMC12549497

[10] Tordjman L, Li JN, Villada G (2025) Non-Healing Nasolabial Ulcerated Plaque: A Clinicopathologic Challenge. Int J Dermatol 64(12):2201–2203. 40817707 10.1111/ijd.70015 PMC12605620

[11] Housley MP, Tillotson S, Parke M, Motaparthi K Cocaine-associated plasma cell orificial mucositis (CAPCOM): an emerging entity. JAAD Case Reports. 2026 Apr 21:S2352-5126(26)00220-1. 10.1016/j.jdcr.2026.04.020. Epub 2026 Apr 21 10.1016/j.jdcr.2026.04.020 PMC13241663 42256370

[12] Trimarchi M, Gregorini G, Facchetti F, Morassi ML, Manfredini C, Maroldi R, Nicolai P, Russell KA, McDonald TJ, Specks U (2001) Cocaine-induced midline destructive lesions: clinical, radiographic, histopathologic, and serologic features and their differentiation from Wegener granulomatosis. Medicine (Baltimore). ;80(6):391–404. 10.1097/00005792-200111000-00005. PMID: 11704715 10.1097/00005792-200111000-00005 11704715

[13] Gill C et al (2023) Cocaine-induced granulomatosis with polyangiitis-an under-recognized condition. Rheumatol Adv Pract 7(1):rkad027. PMID: 37026037; PMCID: PMC10070056 10.1093/rap/rkad027 PMC10070056 37026037

[14] Pendolino AL et al (2024) The role of ANCA in the management of cocaine-induced midline destructive lesions or ENT pseudo-granulomatosis with polyangiitis: a London multicentre case series. Laryngoscope 134(6):2609–2616. Epub 2023 Dec 12. PMID: 38084793 38084793 10.1002/lary.31219

[15] Lood C, Hughes GC (2017) Neutrophil extracellular traps as a potential source of autoantigen in cocaine-associated autoimmunity. Rheumatology (Oxford) 56(4):638–643. PMID: 27354687; PMCID: PMC5850215 27354687 10.1093/rheumatology/kew256 PMC5850215

[16] Coppola N, Cantile T, Canfora F, Adamo D, Bucci P, Mignogna MD, Leuci S (2022) Pitfalls and Challenges in Oral Plasma Cell Mucositis: A Systematic Review. J Clin Med 11(21):6550. PMID: 36362778; PMCID: PMC9659091 36362778 10.3390/jcm11216550 PMC9659091

[17] Brix WK, Nassau SR, Patterson JW, Cousar JB, Wick MR (2010) Idiopathic lymphoplasmacellular mucositis-dermatitis. J Cutan Pathol. ;37(4):426 – 31. 10.1111/j.1600-0560.2009.01371.x. Epub 2009 Jul 15. PMID: 19614724 10.1111/j.1600-0560.2009.01371.x 19614724

[18] Frinculescu A, Parkes HG, Patel F, Shine T, Ramsey J, Frascione N, Abbate V (2026) Analysis of cocaine samples from amnesty bins located at a major UK summer music festival over a 10-year period using both low-field benchtop NMR (60 MHz) and medium-field NMR (400 MHz) for comparison. Forensic Sci Int 384:112902. Epub 2026 Mar 7. PMID: 41819644 41819644 10.1016/j.forsciint.2026.112902

[19] Akhavan A, Alghaithi K, Rabach M, Mirchandani N, Cohen SR (2006) Allergic contact stomatitis. Dermatitis 17(2):88–90 PMID: 16956459 16956459

[20] Roos TC, Merk HF (2001) Allergic contact dermatitis from benzocaine ointment during treatment of herpes zoster. Contact Dermatitis. 44(2):104. 10.1034/j.1600-0536.2001.4402097.x. PMID: 11205383 10.1034/j.1600-0536.2001.4402097.x 11205383

[21] Muratore L, Calogiuri G, Foti C, Nettis E, Di Leo E, Vacca A (2008) Contact allergy to benzocaine in a condom. Contact Dermatitis. 59(3):173-4. 10.1111/j.1600-0536.2008.01359.x. PMID: 18759899 10.1111/j.1600-0536.2008.01359.x 18759899

[22] Wang D, Woo SB (2021) Histopathologic Spectrum of Intraoral Irritant and Contact Hypersensitivity Reactions: A Series of 12 cases. Head Neck Pathol 15(4):1172–1184. Epub 2021 Apr 26. PMID: 33904012; PMCID: PMC8633165 33904012 10.1007/s12105-021-01330-8 PMC8633165

[23] Jego G, Bataille R, Pellat-Deceunynck C (2001) Interleukin-6 is a growth factor for nonmalignant human plasmablasts. Blood. ;97(6):1817-22. 10.1182/blood.v97.6.1817. PMID: 11238125 10.1182/blood.v97.6.1817 11238125

[24] Lee HY, Stieger M, Yawalkar N, Kakeda M (2013) Cytokines and chemokines in irritant contact dermatitis. Mediators Inflamm. ;2013:916497. doi: 10.1155/2013/916497. Epub 2013 Nov 25. PMID: 24371376; PMCID: PMC3858878 10.1155/2013/916497 PMC3858878 24371376

[25] Moreira FP et al (2016) Cocaine abuse and effects in the serum levels of cytokines IL-6 and IL-10. Drug Alcohol Depend 158:181–185. Epub 2015 Nov 26. PMID: 26679059 26679059 10.1016/j.drugalcdep.2015.11.024

[26] Umehara H, Okazaki K, Kawa S, Takahashi H, Goto H, Matsui S, Ishizaka N, Akamizu T, Sato Y, Kawano M (2021) Research Program for Intractable Disease by the Ministry of Health, Labor and Welfare (MHLW) Japan. The 2020 revised comprehensive diagnostic (RCD) criteria for IgG4-RD. Mod Rheumatol 31(3):529–533 Epub 2021 Jan 28. PMID: 33274670 33274670 10.1080/14397595.2020.1859710

[27] Faz-Muñoz D et al (2022) ANCA-associated vasculitis and IgG4-related disease overlap syndrome: a case report and literature review. Immunol Res 70(4):550–559. 35449491 10.1007/s12026-022-09279-8 PMC9023041

[28] Strehl JD, Hartmann A, Agaimy A (2011) Numerous IgG4-positive plasma cells are ubiquitous in diverse localised non-specific chronic inflammatory conditions and need to be distinguished from IgG4-related systemic disorders. J Clin Pathol 64(3):237–243. 21233087 10.1136/jcp.2010.085613

[29] Alabdulaaly L, Jessri M, Treister N, Tavares T, Pettas E, Woo SB, Laubach J, Villa A (2025) Oral plasmacytoma in multiple myeloma patients: report of 18 cases. Oral Surg Oral Med Oral Pathol Oral Radiol 139(6):684–690. 40024819 10.1016/j.oooo.2025.02.002

[30] Whiting C, Schwartzman G, Khachemoune A (2023) Syphilis in Dermatology: Recognition and Management. Am J Clin Dermatol 24(2):287–297. 36689103 10.1007/s40257-022-00755-3 PMC9869822

[31] Sarikhani H, Zhao K, Polacco MA, Gropper C, Helman SN (2024) Benign and malignant cutaneous nasal lesions. Eye ENT Res 1(2):65–91.

[32] Di Maio P, Giudice M, Cavallero A, Carnevale C, Til-Pérez G, Sarría-Echegaray PL, Copelli C, Ramieri G, Iocca O (2025) Head and neck cutaneous basal cell carcinoma: a retrospective analysis of tumour features, surgical margins and recurrences. Eur Arch Otorhinolaryngol 282(6):3183–3191. Epub 2025 Feb 20. PMID: 39979627; PMCID: PMC12122600 39979627 10.1007/s00405-025-09216-z PMC12122600

